# Safety and efficacy of afatinib as add-on to standard therapy of gemcitabine/cisplatin in chemotherapy-naive patients with advanced biliary tract cancer: an open-label, phase I trial with an extensive biomarker program

**DOI:** 10.1186/s12885-018-5223-7

**Published:** 2019-01-11

**Authors:** Markus Moehler, Annett Maderer, Anne Ehrlich, Friedrich Foerster, Arno Schad, Tanja Nickolay, Christian Ruckes, Arndt Weinmann, Visvakanth Sivanathan, Jens U. Marquardt, Peter Robert Galle, Marcus Woerns, Thomas Thomaidis

**Affiliations:** 1 I. Medical Department, Johannes-Gutenberg University, Mainz, Germany; 2 Interdisciplinary Center for Clinical Trials, Johannes-Gutenberg University of Mainz, Mainz, Germany; 3 Department of Pathology, Johannes-Gutenberg University, Mainz, Germany; 4 First Department of Medicine, University Medical Center of the Johannes-Gutenberg University Mainz, Langenbeckstr. 1, 55131 Mainz, Germany

**Keywords:** Cholangiocarcinoma, Afatinib, BIBW 2992, Biomarkers, EGFR, panHER inhibition

## Abstract

**Background:**

To date, the cornerstone of treatment in patients with advanced or metastatic cholangiocarcinoma (CCA) is systemic chemotherapy based on a combination of gemcitabine and a platinum derivative. Other therapeutic approaches including targeted agents and tyrosine kinase inhibitors (TKI) have demonstrated disappointing results, highlighting the complexity of CCA. Recently, drugs aiming at the inhibition of HER-receptors have shown first therapeutic benefit in patients with late stage disease.

The aim of this phase I study was to test the dose level toxicities (DLTs), safety and efficacy of afatinib, a highly specific panErbB family receptor TKI, in chemotherapy naive patients with advanced CCA in conjunction with an extensive biomarker program.

**Methods:**

Afatinib was administered continuously p. o. as add-on in patients with advanced CCA who received conventional chemotherapy with gemcitabine/cisplatin. A classical 3 + 3 phase I study was employed, while the maximum tolerated dose (MTD) of oral afatinib was determined in a 2 step dose escalation. Safety, overall survival (OS) and progression free survival (PFS) were evaluated for all patients. Finally, a translational biomarker analysis was conducted for the EGFR and VEGF signalling cascades.

**Results:**

Overall, 9 patients were enrolled. Further recruitment was discontinued due to lack of efficacy results of the tested drug in other indications.

30 mg afatinib could be safely administered as add-on to 80% of standard dose gemcitabine/cisplatin. The mOS and mPFS were 7.7 and 6.0 months, respectively. Diarrhoea and haematological disorders were the most common observed AEs. Almost all patients overexpressed EGFR on their tumour tissues, whereas none of them expressed mutations in Exons 18, 19 and 21. Non-responders showed a higher variation of VEGF-C, −D, leptin and sEGFR in their sera.

**Conclusions:**

Afatinib failed to show survival benefits in combination with gemcitabine/cisplatin in patients with advanced CCA. Mutational analysis of EGFR and pathways associated with VEGF-C, −D and leptin might show promising results in future studies.

**Clinical trials registration:**

NCT01679405 August, 2012.

**Electronic supplementary material:**

The online version of this article (10.1186/s12885-018-5223-7) contains supplementary material, which is available to authorized users.

## Background

Cholangiocarcinoma (CCA) is the second most common primary liver cancer after hepatocellular carcinoma (HCC) and is characterized by poor prognosis and limited treatment options. Due to the lack of characteristic symptoms, most patients present with advanced-stage disease [1, 2] resulting in a median survival time (mOS) of about six months [3, 4].

Surgical resection remains the only curative treatment for CCA. However, 5-year survival rates vary from 8 to 47% for intrahepatic (iCCA) and 20–54% for extrahepatic (eCCA) biliary cancer due to recurrent or metastatic disease [5, 6]. In patients with advanced, unresectable CCA, systemic chemotherapy has been shown to extend survival compared to best supportive care (BSC). The phase III UK ABC-02 study has established the gemcitabine/cisplatin (gem/cis) combination as a standard of care in these patients [7]. Taking into consideration that the mOS under the current standard regimen is < 1 year [7], new therapeutic approaches for advanced CCA are greatly needed.

The HER family receptors (EGFR/HER1, HER2neu, HER3 and HER4) are associated with increased cellular proliferation, angiogenesis and loss of apoptosis by activation of a complex pathway network including the MAPK kinase cascade, P13K/ATK pathway and STAT transcription factors [8, 9]. A synergy between these receptors - via heterodimerization and cross-linking has been proposed as a mechanism of resistance to single HER inhibitors, suggesting that ErbB family inhibition may overcome the shortcoming of single targeted therapies. In this context, ErbB family inhibitors such as AC480, HM781-36B, HKI-272 and dacomitinib have demonstrated promising results in phase I/II studies in patients with advanced solid tumours [10–12].

The anilino-quinazoline derivative BIBW 2992 (Afatinib, Gilotrif®), is an oral, highly specific, irreversible panErbB family (EGFR, HER2neu, HER4) receptor TKI already approved by the Food and Drug Administration (FDA) and the European Medicines Agency (EMA) for the treatment of non-small-cell lung cancer (NSCLC) [13]. In preclinical animal models, afatinib was safely combined with a variety of chemotherapeutic agents, including paclitaxel, gemcitabine and cisplatin with no significantly increased toxicity [14]. Combination chemotherapy with gemcitabine was also investigated in the Mia-PaCa-2 human pancreatic tumour model [15]. Administration of afatinib did not diminish the efficacy of gemcitabine, but revealed additive anti-tumour activity in this preclinical model of human pancreatic carcinoma [15]. Furthermore, afatinib already demonstrated good tolerability and efficacy as monotherapy in patients with advanced colorectal and esophageal cancer [16, 17].

The aim of this phase I trial was to assess the dose-limiting toxicities (DLTs), the maximum tolerated dose (MTD) as well as the efficacy of afatinib combined with gem/cis in chemotherapy naive patients with advanced CCA. Furthermore, we conducted a thorough translational research analysis of the epidermal growth factor receptor (EGFR) and vascular endothelial growth factor (VEGF) associated cascades to search to identify additional biomarker candidates for further development.

## Methods

### Patients and study design

This was an open-label, uncontrolled phase I trial with a 3 + 3 design evaluating the safety and toxicity as well as the anti-tumour activity of afatinib given as add-on to the combination therapy of gem/cis in chemotherapy naive patients with advanced or metastatic CCA. The investigator-initiated study was conducted at the University Medical Center of the Johannes-Gutenberg University Mainz, Germany. Patients’ enrolment period was 2 years, whereas the last recruited patient began treatment in August 2014. Patients’ inclusion and exclusion criteria are provided as supporting information (Additional file 1: Table S1).

The study was performed in accordance with the provisions of the Declaration of Helsinki, International Conference on Harmonisation, and Good Clinical Practice guidelines (NCT01679405). All patients provided written informed consent before enrolment.

### Treatment and dose-escalation protocol

The MTD of oral afatinib was evaluated in a 2 step dose escalation. The MTD was defined as the highest dose level (DL) at which < 33% of patients experienced a dose-limiting toxicity (DLT, Additional file 2: Table S2). Gem/cis was administered in three week cycles with treatment on day 1 and 8, respectively, whereas afatinib tablets were administered orally continuously (20 mg, 30 mg or 40 mg once daily). In part B of the study it was originally planned to have an expansion cohort with the inclusion of up to seven further patients.

A standard 3 + 3, phase I methodology was employed for each regimen, with three patients being treated at the initial dose (dose level 1). If no DLT was observed within the first cycle in any of these patients, then the dose was escalated to dose level 2 and three more patients were treated. If one patient experienced a DLT, another three patients were treated at the same dose level. In the event that two or more patients experienced a DLT, a dose de-escalation was initiated.

Dose Level 1: 30 mg afatinib, gemcitabine (1.000 mg/m^2^ i. v.)/cisplatin (25 mg/m^2^ i. v.), Dose Level 2: 40 mg afatinib, gemcitabine (1.000 mg/m^2^ i. v.)/cisplatin (25 mg/m^2^ i. v.), Dose Level − 1: 30 mg afatinib, gemcitabine (800 mg/m^2^ i. v.)/cisplatin (20 mg/m^2^ i. v.), Dose Level − 2: 20 mg afatinib, gemcitabine (800 mg/m^2^ i. v.)/cisplatin (20 mg/m^2^ i. v.).

Tumour measurements were done at screening (within day − 30 and day − 1) and subsequently after the second, fourth, sixth and eighth cycle, respectively.

The treatment continued until development of progressive disease according to RECIST 1.1 [18], intolerable toxicity according to the DLT definitions, patient’s decision to discontinue therapy, or up to a maximum of 8 cycles of treatment.

The follow up period was set to 12 months.

Safety was evaluated by the assessment of adverse events (AEs), clinical laboratory tests, vital signs, physical examinations and cardiac function.

### Translational research analysis

#### Immunohistochemistry

Eight of 9 paraffin embedded tissue blocks were available for analysis. Expression of EGFR, HER2neu, HER3, and HER4 were analysed by immunochemistry (IHC).

Three μm thick tissue sections were cut and mounted on super frost slides. These were deparaffinized, rehydrated and peroxidase blocked (3% H_2_O_2_ in methanol, 30 min). Staining was performed on a DAKO autostainer with required staining kits (DAKO) according to the manufacturers’ instructions. Slides were incubated with the respective primary antibodies for EGFR (Clone 2-18C9, DAKO, rtu), HER2neu (polyclonal, DAKO, rtu), HER3 (Clone SP-71, Zytomed, 1:100) and HER4 (polyclonal, Zytomed, 1:50). The staining was evaluated by two independent, blinded investigators as recommended by the manufacture guidelines for membrane staining (DAKO).

#### EGFR mutation analysis

EGFR mutations were analysed in exons 18, 19 and 21 of the EGFR gene after microdissection of FFPE specimens, followed by allele discriminating PCR and Sanger sequencing, according to the manufacturer’s protocol.

#### Enzyme-linked immunosorbent assay (ELISA)

Serum samples of all 9 patients at each cycle on day one were collected and analysed by duo set ELISAs (R&D) for VEGF-A, -C, −D, sEGFR and Hif1α as well as by customized multiplex ELISAs (eBioscience) for EGF, FGF-2, GITR, GTRL, HGF, Leptin, PD-L1, LAG3, VEGF-A and SDF1α on the Bio-Plex MAGPIX System, Bio-Rad. All ELISAs were performed according to the manufacturer’s instructions.

#### Statistical analysis

Biometrical analyses were predefined in the study protocol and its statistical exploration plan was finalized and authorized before start of the clinical trial. The main analysis population was the safety population comprising all patients who received at least one dose of trial treatment. Dose justifications were done according to clinical assessment. For survival parameters including overall survival and time to progression, Kaplan-Meier plots and estimates are presented. Tumour control rates are presented by absolute and relative frequencies.

Due to the small number of patients, descriptive analysis of association between the results of immunohistochemistry, ELISA and clinical-pathological parameters were performed. Comparisons of sera levels and observed changes during therapy were evaluated by Mann Whitney U test.

## Results

### Patient characteristics

A total of 9 patients (pts) were enrolled, of which 3 patients were treated with dose level 1 and 6 patients with dose level − 1. All patients were Caucasians. The median age was 60 years (38–80), and 4 patients (44%) were female (Table 1). 4/5 patients were ECOG 0/1 across dose levels 1 and − 1, respectively. The most common reasons for trial discontinuation were disease progression (*n* = 5, 56%), and AE (*n* = 3, 33%).

**Table 1:** Patients’ demographics

Variable	Dose level 1 (*N* = 3)	Dose level − 1 (*N* = 6)	Total (*N* = 9)
Age [Years]			
N	3	6	9
Mean (SD)	62.00 (21.63)	60.17 (7.31)	60.78 (12.30)
Min	38.0	51.0	38.0
Median	68.00	59.00	60.00
Max	80.0	71.0	80.0
Missing	0	0	0
Gender			
Female	1 (33.33%)	3 (50.00%)	4 (44.44%)
Male	2 (66.67%)	3 (50.00%)	5 (55.56%)
Ethnicity			
Caucasian	3 (100.00%)	6 (100.00%)	9 (100.00%)
Primary tumour			
Intrahepathic tumour	3 (100.00%)	3 (50.00%)	6 (66.67%)
Extrahepatic tumour	0 (0.00%)	3 (50.00%)	3 (33.33%)
ECOG			
0	2 (66.67%)	2 (33.33%)	4 (44.44%)
1	1 (33.33%)	4 (66.67%)	5 (55.56%)
Metastatic disease	3 (100.00%)	6 (100.00%)	9 (100.00%)
Target lesions			
Primary tumour	1 (14.29%)	1 (10.00%)	2 (11.76%)
Liver	3 (42.86%)	3 (30.00%)	6 (35.29%)
Lung	2 (28.57%)	2 (20.00%)	4 (23.53%)
Other lymph nodes	1 (14.29%)	0 (0.00%)	1 (5.88%)
Peritoneum	0 (0.00%)	1 (10.00%)	1 (5.88%)
Soft tissues	0 (0.00%)	1 (10.00%)	1 (5.88%)
Others	0 (0.00%)	2 (20.00%)	2 (11.76%)

*SD* Standard Deviation

Six patients had their primary tumour in the liver, whereas three of them had eCCA. All tumours metastasized and were of grade 2 or 3.

The target lesions were located most frequently in the liver (6 lesions in 6 pts), in mesenterial nodes (2 lesions in 2 pts) and in the lung (4 lesions in 2 pts). The median size of target lesions was 28 mm and ranged from 11 mm to 202 mm. All patients had non-target lesions; they were located most frequently in the liver (7 pts), the lung (7 pts) and in lymph nodes (6 pts. other lymph nodes, 1 pts. lymph nodes of hepatic portal, data not shown).

### Treatment exposure

Study treatment started with three patients in dose level 1. Two of them showed disease progression and stopped study treatment during cycle 7. One patient discontinued treatment due to AEs and did not re-start study treatment, because the interruption lasted longer than allowed by the protocol. Since DLTs appeared in all three patients in dose level 1, the coordinating investigator of the clinical trial decided to decrease the dose of gem/cis proceeding with dose level − 1.

In dose level − 1, diarrhoea and thrombocytopenia occurred in only one patient, therefore three more patients were further treated with the same dose. DLT occurred finally in one out of six patients, and therefore this dose level was defined as the MTD.

In two out of six patients, afatinib dose was reduced to 20 mg per day due to AEs. Two out of six patients in dose level − 1 discontinued treatment due to disease progression. In another patient disease progression was stated at the end of cycle 8. Two patients discontinued due to AEs.

In summary, the mean number of treatment cycles was 4.78 (SD 2.68) with a mean duration of 3.6 months (SD 2.2 months) and a mean number of afatinib doses of 100.89 (SD 60.57, Table 2). The average gemcitabine and cisplatin doses were 797.11 mg/m^2^ (SD 157.31) and 19.89 mg/m^2^ (SD 3.79), respectively.

**Table 2:** Extent of Exposure: Number of cycles, treatment duration and total dose of BIBW 2992 (Safety Population)

	Dose level 1	Dose level − 1	Total
Variable	(*N* = 3)	(*N* = 6)	(*N* = 9)
Number of cycles of chemotherapy			
N	3	6	9
Mean (SD)	5.33 (2.89)	4.50 (2.81)	4.78 (2.68)
Min	2.0	2.0	2.0
Median	7.00	3.50	4.00
Max	7.0	8.0	8.0
Missing	0	0	0
Duration of BIBW Treatment [months]			
N	3	6	9
Mean (SD)	4.0 (2.5)	3.3 (2.3)	3.6 (2.2)
Min	1.3	0.8	0.8
Median	4.4	2.7	3.3
Max	6.3	6.1	6.3
Missing	0	0	0
Total number of BIBW 2992 doses			
N	3	6	9
Mean (SD)	114.33 (53.50)	94.17 (67.54)	100.89 (60.57)
Min	54.0	9.0	9.0
Median	133.00	75.00	79.00
Max	156.0	179.0	179.0
Missing	0	0	0

### Efficacy

**Fig. 1:**
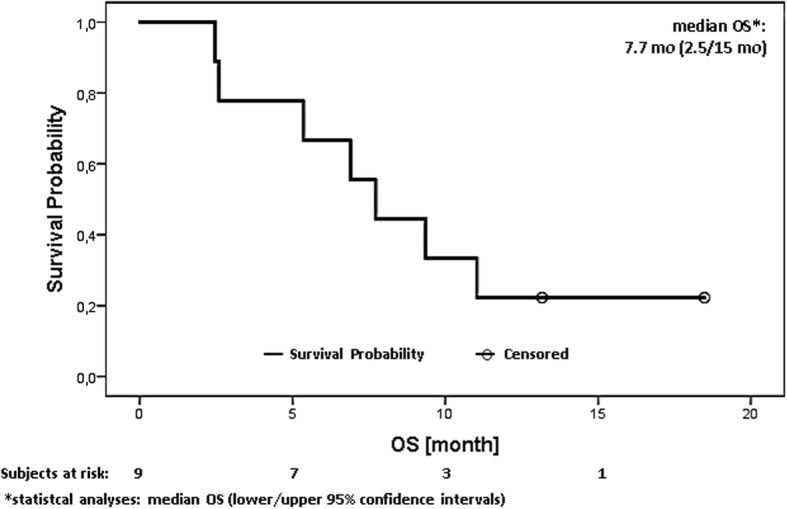
Overall survival analysis in all patients under the combination of BIBW 2992 + gem/cis

**Fig. 2:**
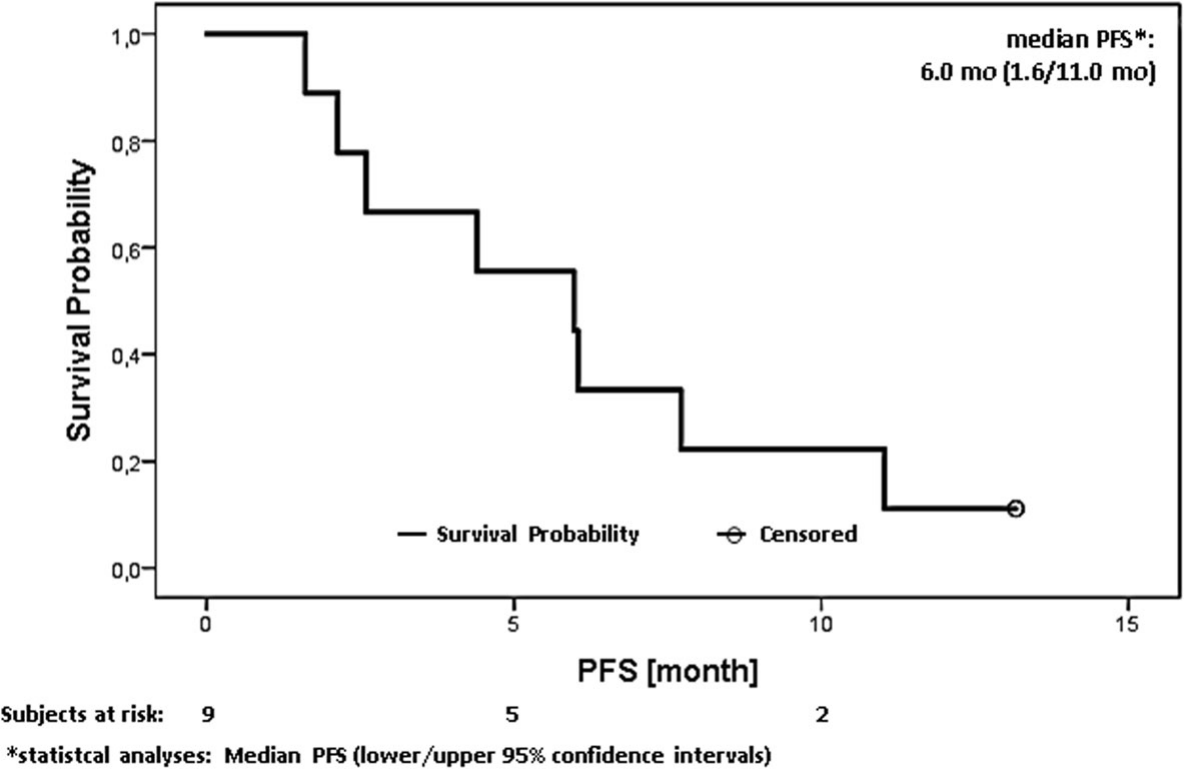
Progression free survival analysis in all patients under the combination of BIBW 2992 + gem/cis

No complete response (CR) and no partial response (PR) according to RECIST 1.1 were observed throughout the study. Four patients had stable disease at the end of treatment and five patients experienced stable disease as the best tumour control. The mOS was 7.7 months (95% CI: 2.5 to 15 Fig. 1) and the median time to progression (mPFS) was 6.0 months (95% CI: 1.6 to 11, Fig. 2). mPFS was defined as the time from start of treatment to first documentation of objective tumour progression according to RECIST 1.1 or a bilirubin level higher than 6 mg/dl as a marker of tumour progression.

### Safety and tolerability

The proportion of patients experiencing treatment-related toxicities are shown in Table 3.

**Table 3:** Adverse Events (Safety Population)

		Dose level 1			Dose level − 1			Total		
	Number of Patients with...	(*N* = 3)		nEvents	(*N* = 6)		nEvents	(*N* = 9)		nEvents
	at least one AE	3	(100.00%)	141	6	(100.00%)	247	9	(100.00%)	388
	SAEs	2	(66.67%)	6	5	(83.33%)	13	7	(77.78%)	19
	Related AEs	3	(100.00%)	102	6	(100.00%)	137	9	(100.00%)	239
	AEs leading to premature study discontinuation	0	(0.00%)	0	1	(16.67%)	1	1	(11.11%)	1
	AEs leading to death	1	(33.33%)	2	1	(16.67%)	1	2	(22.22%)	3

#### Dose limiting toxicities

All three patients in dose level 1 had at least one DLT during treatment. One patient showed hypokalaemia CTC grade III, whereas another had thrombocytopenia CTC grade IV, both during cycle 3 of treatment. The third patient experienced oral mucositis CTC grade III during cycle 1 as well as hypomagnesiaemia CTC grade III and thrombocytopenia CTC grade IV during cycle 2 of treatment.

Only one of six patients in dose level − 1 had DLTs. This patient experienced diarrhoea CTC grade III longer than 7 days and thrombocytopenia CTC grade IV during cycle 2 as well as thrombocytopenia and sepsis both CTC grad IV during cycle 3 of treatment.

#### Serious adverse events (SAEs)

In total, 14 SAEs were reported in all patients. The most common among them were infections, pulmonary embolism, diarrhoea as well as haematological disorders such as anaemia worsening.

#### Adverse events

The most frequent AE in dose levels 1 and − 1, respectively, were fatigue (3/6 pts), nausea (3/5 pts), diarrhoea (2/5 pts), rash (3/4 pts), stomatitis (3/4 pts), anaemia (3/3 pts), weight loss (1/6 pts), epistaxis (3/2 pts), neutropenia (2/3 pts), thrombocytopenia (2/3 pts), hypokalaemia (2/3 pts), paraesthesia (1/4 pts) and paronychia (1/4 pts).

The most frequent Grade 3/4 events (total ≥ 3) were neutropenia (2/3 pts), anaemia (3/1 pts), thrombocytopenia (2/2 pts), diarrhoea (0/3pts) and hypokalaemia (2/1 pts).

### Translational analysis

#### Immunohistochemical staining

**Fig. 3:**
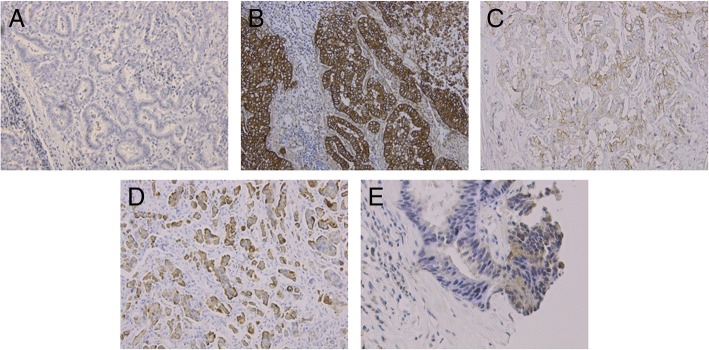
Immunohistochemical staining, **a**) negative control, positive staining of **b**) EGFR (3+), **c**) HER2neu (1+), **d**) HER3 (2+) and **e**) HER4 (only sporadic)

Almost all patients (7/8, 88%) had an overexpression of EGFR on their tumour tissues. The expression of other receptors of the ErbB family (HER2neu, HER3 and HER4) was negative in the majority of the tissue samples (Fig. 3) (three patients showed weak HER2neu, two HER3 and four HER4 membrane staining). There was no correlation for the co-expression of these receptors.

#### Mutational analysis of EGFR by sequencing

The evaluable material (7/9) showed no known EGFR mutations in Exon 18, 19 or 21 in the cohort of afatinib patients.

#### Serum analysis for angiogenesis and other biomarkers

Based on the mOS, patients were subdivided in 2 groups: responders with OS > 7.5 momths (4/9) and non-responders with OS ≤7.5 months (5/9).

**Fig. 4:**
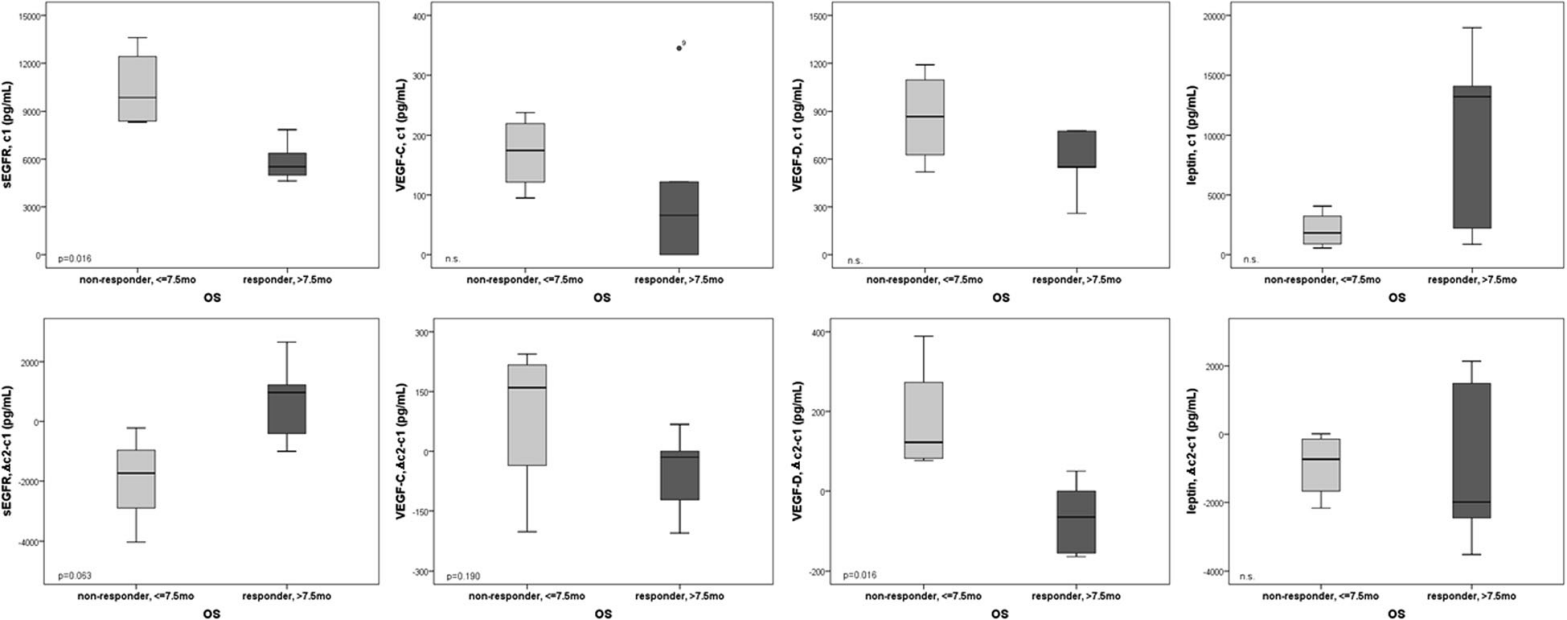
sEGFR, VEGF-C, VEGF-D and leptin serum analysis of non-responders (*n* = 4) OS <=7.5 months and responders (*n* = 5) OS > 7.5 months. Circulated sEGFR, VEGF-C, VEGF-D and leptin levels at baseline (**a-d**). Median changes from the first to the second cycle of treatment of the described markers (**e-h**). Mann-Whitney-U test *p* < 0.05 is determined as significant. n.s. not significant

At the beginning of the therapy, responders had a lower medium serum level of VEGF-C, −D and sEGFR than non-responders. Patients with high leptin serum levels at the start of treatment showed a better response than patients with lower leptin levels. (Fig. 4 a-d).

During therapy (from cycle 1 to cycle 2), responders demonstrated a decrease of VEGF-C, −D and leptin-levels and an increase of sEGFR compared to non-responders (Fig. 4 e-h). Patients with low sEGFR showed a better PFS and OS (*p* = 0.003) than patients with high serum sEGFR (data not shown).

The other examined serum markers showed no correlation with survival (data not shown).

## Discussion

The majority of patients with CCA presents with unresectable disease [19] and their prognosis remains poor. Although chemotherapy has proved beneficial in controlling disease, OS rates of ≥10 months are difficult to achieve, even with triple combinations [20]. Under treatment with cisplatin and gemcitabine, the current standard of care, patients with locally advanced or metastatic disease achieve OS and PFS of 11.7 months and 8 months respectively [7, 21]. Therefore, the focus of recent and current studies remains the identification of a superior treatment combination while minimizing toxicity.

Prospective randomized studies examined the efficacy of single targeted therapies in advanced CCA similarly to treatment strategies for colorectal and gastric cancer. Despite initial encouraging reports [22] the addition of EGFR-inhibiting antibodies such as cetuximab or panitumumab to standard chemotherapy showed no survival benefits in recent randomized trials [23, 24]. Similar results were observed when TKIs targeting VEGF (sorafenib, cediranib) were added to gemcitabine and gemcitabine/cisplatin, respectively [25, 26]. Furthermore, the addition of the TKI erlotinib to gemcitabine and oxaliplatin failed to reach the primary endpoint in a phase III Korean clinical trial [27].

In this phase I study, the panErbB inhibitor afatinib was tested as an add-on to gem/cis in patients with advanced CCA. Administration of 30 mg afatinib on a daily basis was safely combined with a 20% reduced dose of standard gem/cis. Thus, dose level − 1 was defined as MTD and recommended as dose level. This dose reduction seemed to be a rational approach, although 40 mg/d afatinib have already been approved by FDA and EMA for the treatment of NSCLC [13, 28]. However in these cases, afatinib was being administered as monotherapy and not as an add-on to an already potentially toxic regimen such as gem/cis.

In our trial, the most frequent grade 3/4 events were neutropenia, anaemia, thrombocytopenia, diarrhoea and hypokalaemia. Diarrhoea is a common side effect of afatinib already described in the Lux-lung trials [29–31]. The hematologic alterations observed in our study are most likely attributed to the backbone chemotherapy rather than to afatinib itself.

Regarding response to treatment, 63% (5/9) of all evaluable patients showed tumour control for more than 4 months, and four patients achieved stable disease at end of treatment. However, the observed mOS and mPFS (Fig. 1 and Fig. 2) in our study were lower compared with previous key studies [7, 21]. A possible explanation for this reduced anti-tumour activity is that the patient majority received a reduced gem/cis treatment at dose level − 1 in order to avoid cumulative toxicity.

Further analyses in NSCLC revealed that the anti-tumour activity of afatinib is even higher in patients with EGFR mutations in exons 18, 19 and 21 [32, 33]. Although almost all of the patients in our study showed an overexpression of EGFR (Fig. 3) on their tumour tissues, none of the samples in our cohort revealed any of these favourable mutations. These results are in accordance with those published for NSCLC [32, 33]. Although CCA is characterized by different tumour biology, EGFR mutation analysis might be of importance for the choice of therapeutic treatment.

The reports concerning efficacy of ErbB family inhibitors in CCA have so far been restricted to cancer cell lines [34]. Nevertheless, the potential anti-tumour activity of ErbB family inhibition in patients with solid tumours has been at the focus of interest: Dacomitinib and HKI272 are the two most promising drugs that have reached phase III trials in patients with EGFR-mutated NSCLC and HER2neu positive breast cancer, respectively [35, 36]. Dacomitinib has shown superiority over gefitinib as first line treatment in NSCLC patients with Exon 19 or Leu858Arg mutation of EGFR, regarding PFS in the multicenter ARCHER 1050 trial [35]. However, the results concerning OS were inconclusive at data cut off.

Finally, we performed a subgroup analysis with the aim of identifying potentially predictive biomarkers for the treatment with afatinib. As shown in Fig. 4, responders in terms of OS demonstrated lower levels of sEGFR as well as an increase of sEGFR during the first 2 cycles of treatment compared to non-responders. Similar results concerning the potential predictive role of circulated EGFR have been demonstrated in a phase II study with locally advanced esophagogastric cancer [37] as well as in a cohort of 102 NSCLC patients receiving an anti-EGFR therapy [38].

In the case of leptin, we observed an increase of serum levels in patients with no response to afatinib. Leptin has already been associated with the development of CCA through a STAT-3 dependent activation of ERK1/2 [39] and with the development of HCC through angiogenesis [40]. In gastric cancer, leptin induces tyrosine phosphorylation of EGFR resulting in transactivation of this cascade [41]. Our results suggest a complex involvement of leptin in carcinogenesis possibly via cross-linking of different pathways.

Furthermore, the analysis of patient sera in our study showed higher fluctuation of VEGF-C and -D (Fig. 4) but not of VEGF-A (data not shown) between responders and non-responders. These results are in accordance with those of a previously reported phase II trial [42]. In this multicenter study, the addition of bevacizumab to gem/cis did not improve survival compared with historical controls. On the other hand, other studies [43, 44] highlighted the significance of VEGF-C as a prognostic factor in patients with advanced biliary cancer most probably via cross-linking with the nuclear factor (NF)-kB pathway [45].

The limitation of our study is the small number of included patients, since the study was primarily designed to find the MDT and DLTs of afatinib. However, our data do not support the combination treatment of afatinib with gem/cis for advanced CCA as no sufficient clinical or molecular signals for efficacy and subgrouping for treatment selection were observed. Due to the potential inefficacy of EGFR targeting in biliary cancer the sponsor decided not to initiate the originally planned part B and further recruitment of patients in the current trial was discontinued.

## Conclusions

The failure of conventional targeted therapies to improve survival rates in patients with advanced CCA underline the diversity of biliary cancer compared to the rest of gastrointestinal tumours. In this study the ErbB family inhibitor afatinib was safely administered as add-on to 80% of standard dose gemcitabine/cisplatin, but did not show any obvious survival benefits in a small number of patients with advanced CCA. However, the results of recent studies in patients with solid tumours suggest detailed molecular analysis for the identification of a particular group that may benefit from panHer inhibition. In this context, EGFR mutations might play a significant role for treatment stratification similar to NSCLC. Furthermore, other pathways associated with VEGF-C, −D, leptin and sEGFR may play a role in the therapeutic approach to advanced biliary cancer. Nevertheless, further studies are required to investigate the potential of these biomarkers in patients with advanced CCA.

## Additional files

Additional file 1: **Table S1.** Main inclusion and exclusion criteria. Contains the main inclusion and exclusion criteria for patient selection in this study. (DOC 35 kb)

Additional file 2: **Table S2.** Predefined disease limited toxicities. List with the disease limited toxicities associated with afatinib in this study. (DOC 36 kb)

## Abbreviations

(s)EGFR, ErbB: (soluble) Epidermal growth factor receptor
EGF: Epidermal growth factor
FGF: Fibroblast growth factor
GITR: Glucocorticoid-induced TNFR-related protein
HER: Human epidermal growth factor receptor
HGF: Hepatocyte growth factor
HIF1α: Hypoxia-inducible factor 1-alpha
LAG-3: Lymphocyte-activation gene
MAPK: Mitogen-activated protein kinase
PD-L1: Programmed death-ligand 1
PI3K/AKT: Phosphoinositide 3-kinase / Protein kinase B
SDF-1α: Stromal cell-derived factor 1 alpha
STAT: Signal transducer and activator of transcription
VEGF: Vascular endothelial growth factor
