# Supplementary material for: Safety and efficacy of afatinib as add-on to standard therapy of gemcitabine/cisplatin in chemotherapy-naive patients with advanced biliary tract cancer: an open-label, phase I trial with an extensive biomarker program
Source: BMC Cancer. 2019 Jan 11;19:55. doi: 10.1186/s12885-018-5223-7 (PMC6330479; doi:10.1186/s12885-018-5223-7)
Supplement: Supplementary file 1 — Table S1. Main inclusion and exclusion criteria. Contains the main inclusion and exclusion criteria for patient selection in this study. (DOC 35 kb) [file 12885_2018_5223_MOESM1_ESM.doc]

| Main inclusion critera | Main exclusion criteria |
| --- | --- |
| Male and female patients aged ≥ 18 years | Large surgery (except diagnostic biopsy) or smaller surgical procedures, external radiotherapy, brachytherapy, or PDT  within 30 days prior to start of treatment. |
| Signed and dated written informed consent | Other tumour type than adenocarcinoma (e.g. leiomyosarcoma, lymphoma) or a second cancer except for patients with  squamous or basal cell carcinoma of the skin or carcinoma in situ of the cervix which has been effectively treated.  Patients curatively treated and disease free for at least 5 years will be discussed with the sponsor before inclusion |
| Histologically confirmed adenocarcinoma of the gallbladder or intrahepatic bile ducts or extrahepatic bile ducts  (metastasized) or histologically proven hepatic metastases of an earlier resected and histologically proven biliary tract  cancer or a Klatskin tumour (hilar cholangiocarcinoma) not amenable to curative surgical resection | History of acute cardiac disease: congestive heart failure > NYHA class 2; active CAD (MI more than 6 months prior to study entry is allowed); new cardiac arrhythmias requiring anti-arrhythmic therapy (beta blockers or digoxin are permitted); uncontrolled hypertension |
| At least one unidimensionally measurable target lesion in non-irradiated (or treated by photodynamic therapy, PDT) area (largest diameter ≥ 1 cm (spiral CT scan or MRI) | immunosuppressant therapy or with known HIV infection |
| Controlled pain and biliary obstruction | Active clinically severe infections |
| Adequate biliary drainage, no uncontrolled infection | History of organ allograft |
| ECOG Performance Status of 0-1 | Pregnancy |
| LFTs: bilirubin (total) ≤ 1.5 x ULN, ALT/AST/alkaline phosphatase ≤ 2.5 x ULN (≤ 5 x ULN if liver metastases  are present) | Patients with untreated or symptomatic brain metastases. |
| No prior systemic treatment | Prior treatment with EGFR-targeting therapies or treatment with EGFR- or HER2-inhibiting drugs within the past 4  weeks before start of therapy or concomitantly with this trial |
| No prior palliative (radio)-chemotherapy (gem/cis- or fluoropyrimidine based chemotherapy) | Patients unable to comply with the protocol |
|  | Participation in another clinical trial within the past 4 weeks before start of therapy or concomitantly with this trial |

**Table S1. Main inclusion and exclusion criteria**
