# Supplementary material for: Safety and efficacy of afatinib as add-on to standard therapy of gemcitabine/cisplatin in chemotherapy-naive patients with advanced biliary tract cancer: an open-label, phase I trial with an extensive biomarker program
Source: BMC Cancer. 2019 Jan 11;19:55. doi: 10.1186/s12885-018-5223-7 (PMC6330479; doi:10.1186/s12885-018-5223-7)
Supplement: Supplementary file 2 — Table S2. Predefined disease limited toxicities. List with the disease limited toxicities associated with afatinib in this study. (DOC 36 kb) [file 12885_2018_5223_MOESM2_ESM.doc]

| ***Definition of DLT based on CTCAE Version 4.0*** | **Afatinib-related DLTs** |
| --- | --- |
| CTCAE Grade 4 neutropenia that is uncomplicated (not associated with fever > 38.5°C) for > 7 days. | Diarrhoea |
| Neutropenia of any duration associated with fever > 38.5ºC | Nausea, and vomiting. |
| Platelets < 25,000/µl or CTCAE Grade 3 thrombocytopenia associated with bleeding requiring transfusion. | Dehydration and prerenal insufficiency as result of diarrhoea |
| CTCAE Grade 2 or higher decline in cardiac left ventricular function. | Hypokalemia. |
| Persistent CTCAE Grade > 2 diarrhoea for ≥ 7 days despite supportive care. Acute treatment of diarrhoea with e.g. loperamide is allowed. | Mucositis/stomatitis, |
| CTCAE Grade 3 or 4 non-hematologic toxicities (except inadequately treated nausea, untreated vomiting, or untreated diarrhoea). | Rash, acne, pruritus, dry skin, eczema, and folliculitis. |
| For all other DLTs it’s upon the Coordinating Investigator’s discretion whether they are BIBW 2992 or chemotherapy-related. | Fatigue, Anorexia,  . |
| CTCAE Grade 3 or higher worsening of renal function as measured by serum creatinine, newly developed proteinuria, or newly developed decrease in glomerular filtration rate | Interstitial lung disease (ILD) |
| CTCAE Grade > 2 nausea and/or vomiting despite antiemetic treatment. | Pyrexia, Epistaxis |
| All other drug-related non-haematological toxicities of CTCAE Grade ≥ 3, if related to the investigational drug. | Keratitis and ulcerative keratitis, conjunctivitis, dry eye |

**Table S2. Predefined disease limited toxicities**
